# The Reliability Gap: How Traditional Search Engines Outperform Artificial Intelligence (AI) Chatbots in Rosacea Public Health Information Quality

**DOI:** 10.7759/cureus.86543

**Published:** 2025-06-22

**Authors:** Houston C Nelson, Morgan T Beauchamp, April A Pace

**Affiliations:** 1 Dermatology, Eastern Virginia Medical School, Macon and Joan Brock Virginia Health Sciences at Old Dominion University, Norfolk, USA; 2 Public Health, Eastern Virginia Medical School, Macon and Joan Brock Virginia Health Sciences at Old Dominion University, Norfolk, USA; 3 Evidence-Based Medicine, Eastern Virginia Medical School, Macon and Joan Brock Virginia Health Sciences at Old Dominion University, Norfolk, USA

**Keywords:** chatgpt, consumer health information, discern, gemini, generative ai, google, misinformation, readibility, rosacea, search engines

## Abstract

Background: The internet has become a primary source of health information for the public, with important implications for patient decision-making and public health outcomes. However, the quality and readability of this content vary widely. With the rise of generative artificial intelligence (AI) tools such as ChatGPT and Gemini, new challenges and opportunities have emerged in how patients access and interpret medical information.

Objective: To evaluate and compare the quality, credibility, and readability of consumer health information provided by traditional search engines (Google, Bing) and generative AI platforms (ChatGPT, Gemini) using three validated instruments: DISCERN, JAMA Benchmark Criteria, and Flesch-Kincaid Readability Metrics.

Methods: Twenty health-related webpages from each platform were collected using a standardized query across Google, Bing, Gemini, and ChatGPT. Each source was assessed independently by two reviewers using the DISCERN instrument and the adapted JAMA benchmark criteria. Readability was evaluated using the Flesch Reading Ease and Grade Level scores. One-way ANOVA with Bonferroni correction was used to compare platform performance, and Cohen’s Kappa measured inter-rater reliability.

Results: Google achieved the highest mean scores for both quality and credibility (DISCERN: 3.33 ± 0.53; JAMA: 3.70 ± 0.44), followed by Bing, Gemini, and ChatGPT. ChatGPT received the lowest scores across all quality measures. Readability analysis revealed no statistically significant differences between platforms; however, all content exceeded recommended reading levels for public health information. Cohen’s Kappa indicated strong inter-rater agreement across DISCERN items.

Conclusion: Google remains the most reliable source of high-quality, readable health information among the evaluated platforms. Generative AI tools such as ChatGPT and Gemini, while increasingly popular, exhibited significant limitations in accuracy, transparency, and complexity. These findings highlight the need for improved oversight, transparency, and user education regarding AI-generated health content.

## Introduction

The internet has fundamentally transformed how both patients and healthcare professionals access and engage with medical information. This digital shift provides unprecedented access to health education, peer social support networks, and self-management tools. However, the same ease of access also facilitates the rapid spread of low-quality information, often outside the scrutiny of scientific review.

Misinformation refers to false or misleading health content shared without malicious intent, while disinformation denotes deliberately deceptive content. Both have been shown to significantly impact public health, fueling vaccine hesitancy, promoting ineffective treatments, and delaying timely care. The COVID-19 pandemic offered stark examples of how misinformation can undermine public health efforts and fracture trust in medical institutions [1].

While the accessibility of online health content can empower patients, it can also mislead them. For instance, a recent study found that a substantial proportion of patients undergoing elective surgery were unaware of potential risks due to inadequate explanations, often compounded by online misinformation [2]. Another study reported that exposure to misleading content reduced vaccination intentions and increased the prevalence of vaccine-preventable illnesses such as measles [3]. Search engines such as Google, Bing, DuckDuckGo, and Yahoo use complex ranking algorithms that prioritize relevance and popularity, which do not always align with scientific credibility. This sorting can result in the elevation of non-expert sources [4]. Compounding this issue is the lack of transparency in how search results are curated and presented to users, which can obscure the credibility of the sources.

In recent years, the emergence of generative artificial intelligence (AI) platforms, such as ChatGPT and Gemini, has introduced a new paradigm in health information retrieval. These tools generate dynamic, conversational responses, offering users seemingly valid and authoritative answers. However, their outputs are not always grounded in evidence-based medicine, are frequently incomplete or inaccurate, and often lack clear sourcing, potentially amplifying the risks associated with misinformation [5,6]. In some cases, AI tools have presented fabricated citations or outdated guidelines, contributing to confusion among patients and clinicians. Concurrently, studies have shown that younger generations, particularly Generation Z, are increasingly turning to AI tools as primary sources for educational and health information [7]. This shift underscores the importance of critically evaluating the quality of content delivered by these platforms.

Despite this shift in information-seeking behavior, few studies have systematically compared the quality of AI-generated health content with that of traditional search engine results. Head-to-head evaluations using validated assessment frameworks such as DISCERN, JAMA Benchmark Criteria, and Flesch-Kincaid readability measures are lacking. Addressing this gap is essential to guiding both clinical communication and patient education in a digital age.

The objective of this study is to evaluate and compare the quality, credibility, and readability of consumer health information generated by four major platforms: Google, Bing, ChatGPT, and Gemini. Using the DISCERN instrument, JAMA Benchmark Criteria, and Flesch-Kincaid metrics, we aim to assess how these tools support, or hinder, patient understanding of medical conditions and treatment options.

This article was previously presented as a meeting abstract at the Macon & Joan Brock Virginia Health Sciences at Old Dominion University Fine Family Academy of Educators 7th Annual Educational Scholarship Day on May 7, 2025.

## Materials and methods

Study design

This study aims to evaluate the resources commonly used for searching health-related information before or after a diagnosis using freely available, open-access assessment tools, including DISCERN, JAMA Benchmark Criteria, and Flesch-Kincaid metrics [8-10]. The goal is to identify the most reliable sources, those with minimal bias, better organization, and better readability. The authors analyzed the first 20 health-related websites generated in response to search queries in each of four platforms: Google, Bing, Gemini, and ChatGPT 3.5. These platforms were selected due to their widespread and free availability and usage among the public seeking medical information.

To ensure consistency across platforms, a standardized health-related query was used across all search engines and AI tools. The selected query for search engines was “Rosacea,” chosen for its likelihood of yielding consumer-facing content. Each query was conducted in a private browsing window to minimize the influence of prior search history and personalization algorithms. For each platform, the first 20 results were selected for evaluation, reflecting typical user behavior, which shows that most users do not scroll beyond the top results. The query for the AI platforms was "Identify the top 20 rosacea websites." This was done to standardize the resource collection across platforms. The search was run on all platforms on December 4, 2024.

Inclusion and exclusion criteria

Included sources were limited to publicly accessible web pages (non-paywalled) that provided consumer-targeted health information in English. Sponsored content, duplicate links, forums (e.g., Reddit), and user-generated comment sections were excluded. AI-generated summaries (from Gemini and ChatGPT) were recorded directly from the platform interface and were evaluated as discrete entries.

Evaluation tools

DISCERN

DISCERN is an open-access instrument developed by the Centre for Evidence-Based Medicine for judging the quality of written consumer health information on treatment choices. The questionnaire, which is freely available, provides a valid and reliable way for anyone to assess the quality of written information about treatment choices for a health problem [13]. The DISCERN instrument comprises 16 items assessing the quality of consumer health information on treatment choices [8]. Each item is scored on a scale from 1 (low quality) to 5 (high quality), yielding a total possible score of 80. Scores were categorized as follows:

63-80: Excellent: High reliability, comprehensive treatment info

51-62: Good: Generally reliable, may lack minor details

39-50: Fair: Some important elements missing or unclear

27-38: Poor: Serious omissions, bias, or lack of clarity

16-26: Very Poor: Misleading, unreliable, minimally useful content

Two independent raters evaluated each source using DISCERN. The average of the scores was used as the final metric. We tested inter-rater reliability using Cohen’s Kappa.

JAMA Benchmark Criteria

The Journal of the American Medical Association (JAMA) benchmark criteria is a free, open-access tool consisting of four main sections for assessing the quality of journal articles: authorship, attribution, disclosure, and currency [9]. We adapted these criteria for evaluating websites by replacing "journal" with "website or organization" as needed. Each item was scored on a 0-1 basis, for a maximum total score of 4 per source.

A score of 0-1 points indicates insufficient information, a score of 2-3 points indicates partially sufficient information and a score of four indicates sufficient information.

Flesch-Kincaid Readability Metrics

The Flesch-Kincaid readability tests are open-access readability tests designed to indicate how difficult a passage in English is to understand. We evaluated readability using the Flesch Reading Ease Score and Flesch-Kincaid Grade Level instruments [10]. Multiple free online calculators and tools are available to perform Flesch-Kincaid readability assessments, and the formulas are also built into Microsoft Word (Redmond, USA).

Flesch Reading Ease Score (FRES): provides scores between 0 and 100, with higher scores indicating easier-to-read content. A score between 70 and 80 is equivalent to school grade level 8.

The formula for calculating Flesch Reading Ease (FRE) is FRE = 206.835 - (1.015 x ASL) - (84.6 x ASW), where ASL is the average sentence length (words per sentence) and ASW is the average syllables per word.

Flesch-Kincaid Grade Level (FKGL): indicates the U.S. school grade required to comprehend the text. Lower values denote simpler content.

The formula for calculating the FKGL is FKGL = (0.39 x ASL) + (11.8 x ASW) - 15.59.

Statistical analysis

Statistical analysis was conducted using GraphPad Prism 9.0 (GraphPad Software, Inc., Boston, MA, USA). Data were presented as mean ± standard deviation (SD). Differences in DISCERN, JAMA Benchmark, and Flesch-Kincaid ratings among platforms were evaluated using a one-way Analysis of Variance (ANOVA) test followed by Bonferroni-corrected pairwise t-tests with p < 0.05 considered statistically significant. The weighted Cohen’s Kappa coefficient was calculated to assess inter-observer agreement for the DISCERN scores.

## Results

Twenty health information sources per platform were evaluated across Google, Bing, Gemini, and ChatGPT 3.5. Table 3 in the Appendix includes a listing of the URLs from each platform. Each source was assessed independently by two reviewers using the DISCERN and JAMA tools. For the DISCERN tool, we calculated the average score across the 16 items for the final comparison for a maximum score of five. For the JAMA tool, we took the average of the four items for the final comparison for a maximum score of four. Flesch-Kincaid readability metrics were computed using automated analysis tools applied to the body text of each webpage or AI-generated summary.

Quality assessment

DISCERN Scores

Google achieved the highest average DISCERN score across the 16 items (3.33 ± 0.53), indicating the best overall quality of health information. Bing followed (3.13 ± 0.91), with Gemini (2.67 ± 0.87) and ChatGPT (2.20 ± 1.32) trailing, suggesting poorer information quality and reliability. Google outperformed all others in 12 out of 16 DISCERN categories (Appendix).

JAMA Benchmark Scores

Google led again with a mean score of 3.7 ± 0.44, followed by Bing (3.48 ± 0.92), Gemini (3.15 ± 1.15), and ChatGPT (2.38 ± 1.44). ChatGPT’s lower performance reflects deficiencies in authorship and attribution transparency.

Readability assessment

Flesch Reading Ease Scores

Although Google had the highest mean readability score (44.92 ± 20.54), no statistically significant differences were found among platforms (p = 0.2085). All platforms scored below 50, suggesting that their content may be difficult for the public to comprehend.

Flesch-Kincaid Grade Levels

Google content was closest to the high school reading level (11.61 ± 4.79), while Gemini (15.36 ± 7.10) required the highest literacy level. Differences were not statistically significant (p = 0.1995).

Notably, none of the evaluated platforms produced content that met the American Medical Association’s (AMA) or National Institute of Health’s (NIH) recommended 6th-8th grade reading level for public health materials [11]. This suggests a potential barrier to comprehension for individuals with lower health literacy and underscores the need for more accessible online content.

Table 1 presents the mean scores (± standard deviation) for DISCERN, JAMA Benchmark Criteria, and Flesch-Kincaid Readability Metrics across search platforms (Google, Bing, Gemini, and ChatGPT).

**Table 1:** Mean quality and readability scores for all websites

Mean Quality and Readability Scores for all Websites (Mean score ± SD)				
	DISCERN	JAMA	F-K Reading Ease	F-K Grade Level
Google	3.33125 ± 0.53	3.7 ± 0.44	44.91535 ± 20.54	11.6102 ± 4.79
Bing	3.13281 ± 0.91	3.475 ± 0.92	33.62375 ± 21.98	13.62405 ± 5.21
Gemini	2.66719 ± 0.87	3.15 ± 1.15	33.55305 ± 21.81	15.35835 ± 7.10
ChatGPT	2.20312 ± 1.32	2.375 ± 1.44	31.2635 ± 23.90	13.9961 ± 4.52

While mean scores are informative, substantial variability was observed within certain platforms. For example, ChatGPT responses ranged from high-quality summaries with near-perfect DISCERN scores to others lacking citation or treatment risk information. This inconsistency may reflect variability in prompt interpretation or model behavior, further complicating user trust.

Statistical significance

To evaluate differences among the four platforms, a one-way ANOVA was conducted to assess variation between the sample means. The differences were found to be statistically significant for the DISCERN (0.000000961) and JAMA (0.00096) scores, prompting pairwise t-tests to identify which platform comparisons were significant. To control multiple comparisons and reduce the risk of Type I error, the Bonferroni correction was applied. Significant differences were found for the DISCERN Google-Gemini (p=0.00009), Google-ChatGPT (p=0.0000033), and Bing-ChatGPT (p=0.0004461) comparisons. For the JAMA scores, pairwise t-tests with Bonferroni correction found significant differences for the Google-ChatGPT (p=0.0003390) and Bing-ChatGPT (0.0006551) comparisons. The results of all pairwise comparisons are detailed in Table 2.

**Table 2:** p-values for ANOVA with pairwise comparisons: DISCERN, JAMA Benchmark (JAMA), Flesch Reading Ease (FK RES), and Flesch Kincaid Grade Level (FK GL) instruments. Shaded comparisons are statistically significant. Bonferroni correction was used for each instrument (.05/6 = 0.0083).

ANOVA p-value	Google vs. Bing	Google vs. Gemini	Google vs. ChatGPT 3.5	Bing vs. Gemini	Bing vs. ChatGPT 3.5	Gemini vs. ChatGPT 3.5
DISCERN 0.000000961	0.2359717	0.0000900	0.0000033	0.0215219	0.0004461	0.0670457
JAMA 0.00096	0.3321709	0.0532242	0.0003390	0.3311039	0.0006551	0.0676104
FK RES 0.208528	0.1014342	0.0988060	0.0601769	0.9919056	0.7468954	0.7533865
FK GL 0.199525	0.2111295	0.0577078	0.1136081	0.3841049	0.8108024	0.4736583

Cohen’s Kappa (κ) was used to measure inter-rater agreement in DISCERN scores. The agreement ranged from a low of 0.56 (indicating moderate agreement) to a perfect score of 1.0. Over 81% of the 64 paired ratings were in the perfect/near-perfect range. These values demonstrate strong agreement across most categories, with the highest reliability observed in items addressing clarity of treatment choices and transparency of sources.

## Discussion

This study addresses a critical and timely issue at the intersection of health communication, digital literacy, and artificial intelligence. As generative AI platforms become embedded into everyday search behaviors, patients are increasingly exposed to health information that may lack scientific rigor, transparency, or accessibility. While search engines like Google have long served as gatekeepers to health information, AI tools now generate fluent and persuasive content that may not be grounded in evidence-based medicine [6].

Our data provides valuable insight into the current landscape of digital health information access and quality. Google remains the largest and most widely used search engine, and with the integration of generative AI such as Gemini into its results, it is crucial to understand how these tools influence patient behavior and understanding. Although our findings suggest that Google delivers the highest quality and most readable content overall, the integration of generative AI summaries may affect how users interact with information, most users are likely to engage with the top-of-page AI-generated content before exploring traditional links.

Both ANOVA analyses and overall mean comparisons showed Google to be the best-performing search engine and Gemini to be the strongest generative AI tool in this cohort. However, Gemini’s performance still lagged behind Bing in some domains, indicating potential issues with information accuracy and transparency. Based on these results, we recommend that clinicians guide their patients to use traditional Google search results over AI summaries and to rely on AI-generated content, such as Gemini or ChatGPT, only for general background knowledge, not for clinical decision-making.

These findings contribute to the broader discourse on digital health information-seeking behavior. Models such as the Health Belief Model and the Theory of Planned Behavior suggest that patients’ perceptions of trustworthiness, accessibility, and relevance influence how they use health information [12]. Our results indicate that users often favor AI tools and high-ranking search results, potentially due to cognitive heuristics that associate visibility with credibility. This behavior, while understandable, may lead to overreliance on tools that present oversimplified or incomplete data.

As patients increasingly arrive at medical encounters armed with information gathered online, clinicians are shifting from being the primary source of knowledge to interpreters and validators of digital content. This dynamic creates new opportunities and challenges, while some patients may be more engaged and proactive, others may hold misconceptions influenced by biased or inaccurate AI-generated summaries. To meet this challenge, future medical education should train providers to address digital misinformation, validate credible information, and manage AI-influenced patient expectations.

Generative AI tools, while powerful, frequently lacked transparency in both sourcing and methodology. ChatGPT often produced content that cited a limited set of sources or included broken or irrelevant links. This repetition and lack of diversity in sources raise serious concerns about algorithmic bias and the potential for reinforcing misinformation. Moreover, there is little to no disclosure within AI interfaces regarding the underlying databases or training sources, leaving users unable to critically assess the trustworthiness of the content. Without transparent sourcing, AI-generated information poses ethical challenges, especially in healthcare settings where accuracy, balance, and informed decision-making are paramount.

Our findings align with existing literature demonstrating that online health content often exceeds recommended readability levels. The AMA and NIH recommend that health information be written at a 6th- to 8th-grade reading level to ensure accessibility [11]. However, the content analyzed from all platforms in our study required high school to college-level literacy. This presents a major barrier to patient comprehension and equitable access to information. Tools like Gemini and ChatGPT produced some of the most complex and jargon-heavy websites, further distancing them from the informational needs of the average patient.

These findings offer actionable implications for public health communication. Search engines and AI developers should consider integrating readability filters, evidence-based citations, and plain-language summaries to support better comprehension and informed decisions. Partnerships between public health agencies and technology companies could help elevate reliable information within search rankings and mitigate the influence of low-quality or AI-generated misinformation.

Moreover, the study underscores the need for ethical design and regulation of AI tools used in health communication. Developers should adopt guidelines for content transparency, bias mitigation, and regular auditing to ensure that generative AI platforms are safe and trustworthy sources of medical information. Public policy efforts may also be needed to establish regulatory frameworks governing the use of AI in health communication, particularly as adoption continues to grow.

Finally, this study opens the door for future research into how users interpret and act upon AI-generated health content. Longitudinal and behavioral studies are needed to assess the real-world consequences of using search engines and generative AI as primary sources of health information. Understanding the impact on patient outcomes, decision-making, and provider trust will be essential for shaping future digital health strategies.

Limitations

This study had several limitations, notably the modification of evaluation criteria to better align with our specific research objectives. While altering the JAMA criteria may have been necessary for our analysis, it introduces the potential for bias, particularly in the way the content was assessed. To minimize this risk, we made efforts to compare scores across evaluators. In future studies, a larger and more diverse group of participants in the DISCERN evaluation process would be beneficial to further reduce bias and improve the reliability of the findings.

Additionally, our analysis focused exclusively on text-based results. Many platforms increasingly include visual content, multimedia explanations, and interactive tools that can influence how users interpret health information. The exclusion of these elements limits the generalizability of our findings.

Another important consideration is the dynamic and rapidly evolving nature of both search engine algorithms and generative AI models. The results presented in this study represent a snapshot in time and may not reflect future iterations of these platforms. As developers make frequent updates to improve or optimize their models, future analyses will be necessary to assess changes in performance and quality.

Furthermore, the scope of search queries was limited to a specific health condition. A broader variety of clinical topics, particularly chronic conditions, rare diseases, or public health content may yield different results. Future research should include more diverse health domains and explore multilingual queries to evaluate cross-cultural accessibility.

While inter-rater reliability was strong, qualitative assessment tools such as DISCERN and JAMA are inherently subject to interpretation. Although we used multiple reviewers and Cohen’s Kappa to mitigate bias, the inclusion of more diverse raters, including laypeople and healthcare professionals from various specialties, would enhance the robustness of future evaluations.

## Conclusions

Our analysis indicates that Google remains the most reliable search engine for obtaining rosacea-related health information, as evidenced by the breadth and quality of sources available. However, the rise of generative AI technologies, such as ChatGPT and Gemini, signals a shift in how health information is accessed. While Gemini performed better than ChatGPT in this study, generative AI platforms can be valuable for providing broad overviews or filling knowledge gaps, but they are not yet suitable as the primary source of medical information. Despite growing popularity, generative AI tools displayed notable deficiencies in content quality, transparency, and readability. ChatGPT showed inconsistent attribution and complex language that may hinder accessibility for the average reader. While generative summaries, like those offered by Gemini, may serve as a starting point for patient inquiry, they should be interpreted cautiously and supplemented with evidence-based information. These findings underscore the need for clinicians to play a proactive role in educating patients about how to identify and use reliable online health information. Incorporating digital health literacy into clinical care and public health campaigns could empower patients to navigate the digital landscape more critically and avoid misinformation.

From a development and policy perspective, the results suggest a need for improved transparency and oversight of AI-generated medical content. Developers of AI tools should integrate evidence-based sourcing, bias mitigation strategies, and clear disclosures into their platforms. Policymakers may also consider establishing quality standards or regulatory frameworks to ensure the safe dissemination of health information online. Search engines and AI tools should also consider incorporating features such as quality indicators, readability filters, and plain-language summaries to help users more easily evaluate content credibility. Such enhancements could significantly improve user comprehension and promote informed medical decision-making. Finally, this study highlights a critical need for longitudinal and behavioral research on the use of generative AI in health contexts. Understanding how individuals engage with and act upon AI-generated content will be essential in determining the broader public health implications of these tools. While Google currently offers the most trustworthy and accessible health information, the integration of AI technologies is reshaping the landscape. Continuous evaluation and responsible innovation will be vital to ensuring that these evolving tools support, rather than undermine, patient health and decision-making.

## Appendices

**Table 3:** Top 20 URLs from each platform

Top 20 Websites: Platforms searched 12/4/2024	
Google	
1	https://www.mayoclinic.org/diseases-conditions/rosacea/symptoms-causes/syc-20353815
2	https://www.niams.nih.gov/health-topics/rosacea
3	https://www.rosacea.org/patients/all-about-rosacea
4	https://my.clevelandclinic.org/health/diseases/12174-rosacea
5	https://www.webmd.com/skin-problems-and-treatments/understanding-rosacea-basics
6	https://www.aad.org/public/diseases/rosacea
7	https://en.wikipedia.org/wiki/Rosacea
8	https://www.hopkinsmedicine.org/health/conditions-and-diseases/rosacea
9	https://www.rosacea.org/
10	https://www.nhsinform.scot/illnesses-and-conditions/skin-hair-and-nails/rosacea/
11	https://www.aad.org/public/diseases/rosacea/triggers/prevent
12	https://dermnetnz.org/topics/rosacea
13	https://www.betterhealth.vic.gov.au/health/conditionsandtreatments/rosacea
14	https://medlineplus.gov/ency/article/000879.htm
15	https://www.webmd.com/skin-problems-and-treatments/skin-conditions-rosacea
16	https://www.cedars-sinai.org/health-library/diseases-and-conditions/r/rosacea.html
17	https://familydoctor.org/condition/rosacea/
18	https://www.medicalnewstoday.com/articles/160281
19	https://www.ncbi.nlm.nih.gov/books/NBK557574/
20	https://www.healthline.com/health/skin/rosacea
Bing	
1	https://www.mayoclinic.org/diseases-conditions/rosacea/symptoms-causes/syc-20353815
2	https://www.rosacea.org/patients/all-about-rosacea
3	https://www.rosacea.org/
4	https://health.clevelandclinic.org/rosacea-natural-treatments
5	https://www.aad.org/public/diseases/rosacea/treatment/diagnosis-treat
6	https://www.aafp.org/pubs/afp/issues/2024/0600/rosacea.html
7	https://www.aad.org/public/diseases/rosacea/insider/things-to-know
8	https://www.aad.org/public/diseases/rosacea/what-is
9	https://www.niams.nih.gov/es/informacion-de-salud/rosacea
10	https://www.ncbi.nlm.nih.gov/books/NBK557574/
11	https://www.yalemedicine.org/conditions/rosacea
12	https://www.healthline.com/health/skin/rosacea
13	https://www.aad.org/public/diseases/rosacea/what-is/causes
14	https://www.healthline.com/health/rosacea-natural-treatments
15	https://www.nhs.uk/conditions/rosacea/
16	https://medlineplus.gov/spanish/rosacea.html
17	https://www.niams.nih.gov/es/informacion-de-salud/rosacea
18	https://www.ncbi.nlm.nih.gov/books/NBK557574/
19	https://www.yalemedicine.org/conditions/rosacea
20	https://www.hopkinsmedicine.org/health/conditions-and-diseases/rosacea
Gemini	
1	National Rosacea Society: https://www.rosacea.org/
2	MedlinePlus - Rosacea: https://medlineplus.gov/ency/article/000879.htm
3	Rosacea Resource Center - American Academy of Dermatology: https://www.aad.org/public/diseases/rosacea
4	Healthline - Rosacea: https://www.healthline.com/health/video/rosacea-triggers
5	Verywell Health - Rosacea: https://www.verywellhealth.com/rosacea-overview-4801740
6	Mayo Clinic - Rosacea: https://www.mayoclinic.org/diseases-conditions/rosacea/diagnosis-treatment/drc-20353820
7	Cleveland Clinic - Rosacea: https://www.theparkerclinic.com/skin-aesthetic-treatments-cleveland/skin-concerns/rosacea/
8	WebMD - Rosacea: https://www.webmd.com/skin-problems-and-treatments/understanding-rosacea-basics
9	HealthCentral - Rosacea: https://www.skincentral.com/en/rosacea/
10	MedicineNet - Rosacea: https://pubmed.ncbi.nlm.nih.gov/12182520/
11	John Hopkins Medicine - Rosacea: https://www.hopkinsmedicine.org/health/conditions-and-diseases/rosacea
12	University of Michigan - Rosacea: https://medicine.umich.edu/dept/cosmeticderm/treated-conditions/facial-redness-rosacea
13	University of California, San Francisco - Rosacea: https://clinicaltrials.ucsf.edu/rosacea
14	Stanford Health Care - Rosacea: https://stanfordhealthcare.org/medical-conditions/skin-hair-and-nails/acne-rosacea.html
15	Mount Sinai - Rosacea: https://www.mountsinai.org/care/dermatology/services/rosacea
16	New York University Langone Health - Rosacea: https://nyulangone.org/conditions/rosacea/support
17	The American Journal of Clinical Dermatology - Rosacea: https://link.springer.com/journal/40257
18	The Journal of Clinical and Aesthetic Dermatology - Rosacea: https://jcadonline.com/diagnosing-treating-rosacea-skin-of-color/robinson_figure1-2/
19	The British Journal of Dermatology - Rosacea: https://academic.oup.com/bjd/article/179/2/282/6730927
20	The Journal of Investigative Dermatology - Rosacea: https://www.jidonline.org/
ChatGPT 3.5	
1	American Academy of Dermatology (AAD): Rosacea Overview
2	National Rosacea Society (NRS): National Rosacea Society
3	Mayo Clinic: Mayo Clinic - Rosacea
4	WebMD: WebMD - Rosacea
5	Healthline: Healthline - Rosacea
6	Cleveland Clinic: Cleveland Clinic - Rosacea
7	DermNet: DermNet - Rosacea
8	NHS (National Health Service, UK): NHS - Rosacea
9	Verywell Health: Verywell Health - Rosacea
10	Medical News Today: Medical News Today - Rosacea
11	American Academy of Dermatology Association: AAD Association - Rosacea
12	The Skin Cancer Foundation: Skin Cancer Foundation - Rosacea
13	Johns Hopkins Medicine: Johns Hopkins - Rosacea
14	Cleveland Clinic Health Essentials: Cleveland Clinic Health Essentials - Rosacea
15	American Society of Plastic Surgeons: American Society of Plastic Surgeons - Skin Issues
16	WebMD - Rosacea Treatment Overview: WebMD - Rosacea Treatment Overview
17	Health Magazine: Health Magazine - Rosacea Guide
18	PubMed Health: PubMed Health - Rosacea
19	Everyday Health: Everyday Health - Rosacea
20	Consumer Reports - Rosacea: Consumer Reports - Rosacea

**Table 4:** DISCERN Quality Criteria Scores for Rosacea Websites

Quality criteria	Mean score ± SD			
	Google	Bing	ChatGPT	Gemini
1. Are the aims clear?	4.8 ± 0.89	4.4 ± 1.02	2.9 ± 2.02	4.025 ± 1.73
2. Does it achieve its aims?	4.775 ± 1.23	4.4 ± 1.46	2.9 ± 2.02	4.025 ± 1.73
3. Is it relevant?	4.8 ± 0.89	4.4 ± 1.91	2.9 ± 2.02	4.15 ± 1.63
4. Is it clear what sources of information were used to compile the publication (other than the author or producer)?	4.2 ± 1.35	4.1 ± 1.89	2.75 ± 1.96	3.35 ± 1.81
5. Is it clear when the information used or reported in the publication was produced	2.75 ± 1.4	3.275 ± 1.54	2.15 ± 1.41	2.475 ± 1.6
6. Is it balanced and unbiased?	4.3 ± 1.23	3.8 ± 1.93	2.825 ± 1.99	3.475 ± 1.54
7. Does it provide details of additional sources of support and information?	3.05 ± 1.7	2.225 ± 1.55	1.875 ± 1.4	1.7 ± 1.3
8. Does it refer to areas of uncertainty?	1.275 ± 0.64	1.225 ± 0.49	1.025 ± 0.16	1.325 ± 0.81
9. Does it describe how each treatment works?	4.275 ± 1.48	3.6 ± 1.94	2.7 ± 1.94	3 ± 1.92
10. Does it describe the benefits of each treatment?	3.175 ± 1.76	3.225 ± 1.76	2.15 ± 1.56	2.375 ± 1.75
11. Does it describe the risks of each treatment?	1.375 ± 0.71	1.875 ± 1.09	1.35 ± 0.69	1.375 ± 0.78
12. Does it describe what would happen if no treatment was used?	1.3 ± 0.91	1.6 ± 0.83	1 ± 0.0	1.1 ± 0.45
13. Does it describe how the treatment choices affect overall quality of life?	2.05 ± 1.15	1.725 ± 0.97	1.35 ± 0.74	1.3 ± 0.52
14. Is it clear that there may be more than one possible treatment choice?	5 ± 0.0	4.4 ± 1.91	2.9 ± 2.02	3.5 ± 1.97
15. Does it provide support for shared decision-making?	2.525 ± 1.35	2.5 ± 1.28	2.1 ± 1.44	2.35 ± 1.47
16. Based on the answers to all of the above questions, rate the overall quality of the publication as a source of information about treatment choices.	3.425 ± 1.02	3.35 ± 1.5	2.375 ± 1.52	2.75 ± 1.07
